# Huntingtin and Other Neurodegeneration-Associated Proteins in the Development of Intracellular Pathologies: Potential Target Search for Therapeutic Intervention

**DOI:** 10.3390/ijms232415533

**Published:** 2022-12-08

**Authors:** Aleksandra S. Churkina (Taran), Anton S. Shakhov, Anatoly A. Kotlobay, Irina B. Alieva

**Affiliations:** 1 A.N. Belozersky Institute of Physico-Chemical Biology, Lomonosov Moscow State University, 1–40, Leninskye Gory, 119992 Moscow, Russia; 2 Faculty of Bioengineering and Bioinformatics, Lomonosov Moscow State University, 1–73, Leninskye Gory, 119992 Moscow, Russia; 3 Lopukhin Federal Research and Clinical Center of Physical-Chemical Medicine of Federal Medical Biological Agency, 1a Malaya Pirogovskaya St., 119435 Moscow, Russia

**Keywords:** neurodegeneration, neurodegenerative diseases, polyglutamine diseases, proteinopathies, Huntington’s disease, huntingtin

## Abstract

Neurodegenerative diseases are currently incurable. Numerous experimental data accumulated over the past fifty years have brought us closer to understanding the molecular and cell mechanisms responsible for their development. However, these data are not enough for a complete understanding of the genesis of these diseases, nor to suggest treatment methods. It turns out that many cellular pathologies developing during neurodegeneration coincide from disease to disease. These observations give hope to finding a common intracellular target(s) and to offering a universal method of treatment. In this review, we attempt to analyze data on similar cellular disorders among neurodegenerative diseases in general, and polyglutamine neurodegenerative diseases in particular, focusing on the interaction of various proteins involved in the development of neurodegenerative diseases with various cellular organelles. The main purposes of this review are: (1) to outline the spectrum of common intracellular pathologies and to answer the question of whether it is possible to find potential universal target(s) for therapeutic intervention; (2) to identify specific intracellular pathologies and to speculate about a possible general approach for their treatment.

## 1. Introduction

Neurodegenerative diseases are an extensive group of human pathologies of various genesis, among which both hereditary diseases and pathologies developed de novo are described. These diseases develop over decades, so they are more common in older people. In many countries of the world in recent decades, especially in countries with developed economies, in parallel with the increase in average life expectancy there has been an increase in the number of neurodegenerative diseases. They have become one of the main problems that worsen the quality of life in old age. Despite the difference in clinical manifestations, the development of neurodegenerative diseases may be based on similar molecular and/or cellular mechanisms. Considerable attention has been paid to the elucidation of these mechanisms in the last fifty years, and significant progress has been made in understanding the causes of their occurrence at the molecular, cellular and organic levels. However, this knowledge is still insufficient not only for the prevention, but also for the treatment of neurodegenerative diseases. In addition, if we do not talk about hereditary or genetically determined pathologies, there is also a problem of diagnosis and early detection of sporadically developing neurodegenerations.

The most common and actively researched neurodegenerative diseases are Alzheimer’s (AD) and Parkinson’s (PD) diseases. There is a wide range of less common pathologies, including hereditary causes. The classification of these diseases can be based on various principles: by clinical manifestations (dementia, tremor, chorea, etc.), by anatomical features (atrophy of various brain parts), as well as by the nature of molecular disorders and cellular pathologies underlying the disease development [1,2]. Classification based solely on molecular data is often inapplicable in clinical practice, since it is difficult to determine the nature of cellular disorders at the beginning of the disease development. Therefore, the most accepted classification at the moment is the combined classification of neurodegenerative diseases, based on a combination of clinical manifestations of the disease and analyses of biochemical modifications of proteins, as well as the nature of their accumulation. In addition, anatomical and cellular disorders are considered [3,4].

At the moment, it is known that the basis of neurodegenerations are proteinopathies, disorders in the structure and function of various proteins that lead to their aggregation and toxic effects on cells. The most common neurodegenerative proteinopathies are amyloidosis (amyloid extracellular plaques in AD), tauopathy (various dementias), α-synucleopathy (Lowy bodies in PD), prionopathy, and TDP-43 proteinopathy (in amyotrophic lateral sclerosis (ALS)). Among hereditary neuropathologies, a group of polyglutamine diseases can be distinguished caused by the repetition of the cytosine-adenine-guanine (CAG) trinucleotide in the coding segment of certain genes, which leads to the appearance of mutant proteins. Huntington’s disease (HD) is one of the best studied polyglutamine diseases [5].

In addition, it is known that with the development of some neurodegenerative diseases, the accumulation of several different proteins is observed. Simultaneous accumulation of tau protein and amyloid-β (Aβ) protein in AD [6], Aβ and α-synuclein in PD and dementia [7,8], complicates their clinical interpretation. In these cases, the concept of multiproteinopathies is required. Abnormal proteins may have a well-defined origin, determined by a specific mutation, as in the case of HD [5], or of an uncertain nature, as in the cases of sporadic forms of AD and PD.

The greatest difficulty for the timely clinical diagnosis of neurodegenerative diseases is their sporadic forms. They are associated with a variety of genetic factors and environmental influences. The causes of their occurrence remain largely unknown. In general, the development of sporadic forms of neurodegenerative diseases leads to the same cellular pathologies as in hereditary forms, including the accumulation of toxic proteins. Modern scientific research is aimed at identifying specific biomarkers that would select potential patients in the early stages of the disease before the first symptoms appear. Determining the risk group is of great importance, because now methods have been developed to slow down the development of the disease, and the earlier such therapy begins, the better for the patient. For instance, in a number of publications devoted to this problem, an increased level of methylation of risk genes associated with the development of neurodegenerative diseases is discussed [9,10,11].

In all neurodegenerative diseases, similar cellular pathologies are observed, among which are listed the accumulation of protein aggregates, violation of protein proteolysis, mitochondrial disorders, transport disorders (including neurotransmitters), changes in protein expression, and others. A question naturally arises: which processes accompanying neurodegeneration are the consequence of the cellular pathology development, and which are the cause? Neurodegenerations often develop according to a similar scenario at the tissue and organizational level. However, from a practical point of view, it is important to understand whether it is possible to identify intracellular disorders responsible for the development of several neurodegenerative diseases. This issue is often the subject of discussion during scientific conferences and round tables, and the purpose of such discussions is to try to identify, at least theoretically, intracellular targets for creating a general targeted therapy. Identifying possible targets may provide a key to the neurodegeneration’s treatment. Nevertheless, the fundamental question remains open: whether it is possible to find a common approach to the treatment of all (or at least a group of) neurodegenerative diseases, or an individual approach is required for each disorder? In this review we will try to answer this question by considering both widespread intracellular pathologies characteristic of many neurodegenerative diseases and unique ones peculiar only to a certain disease.

## 2. Proteins Responsible for Neurodegenerative Diseases, Their Association with Cell Organelles and Involvement in Cellular Functions

Most neurodegenerative diseases are proteinopathies that occur as a result of disorders in the structure and function of certain proteins. These defective proteins disorganize the normal function of intracellular organelles, which ultimately leads to toxic damage to nerve cells.

### 2.1. Amyloid-β

AD is one of the most common and severe neurodegenerative diseases. It is considered to be proven that AD is amyloidosis by nature; the disease is characterized by the formation of extracellular amyloid plaques in the nervous tissue. It is believed that their accumulation leads to the development of neurodegenerative disorders in patients, however (and we cannot fail to note this), these long-established ideas can be revised [12].

It has been shown that Aβ is formed by the cleavage of Aβ precursor protein (APP) by β- and γ-secretase. A number of β-amyloid peptides with a length of 39 to 43 amino acid residues are formed, the hydrophobic nature of which promotes self-aggregation and neurotoxicity [13,14]. A series of conformational changes in Aβ eventually leads to the deposition of amyloid plaques, which is facilitated by a violation of the balance between the formation and cleavage of Aβ. It is assumed that plaques with Aβ accumulate in patients with AD precisely in the extracellular space [13,14,15]. Aβ is also secreted by normal cells in culture and is found as a circulating peptide in the plasma and cerebrospinal fluid (CSF) of healthy humans and other mammals [16,17,18]. When this fact was established, it turned out that some other fragments of APP are also secreted by cells during normal metabolism [19].

APP is an expressed transmembrane protein (reviewed in [20,21]) that appears to perform important physiological functions in the synapse. It has been suggested that APP may function as a receptor involved in transsynaptic signaling [22,23], and may also participate in adhesion [24,25]. Current data clearly indicate an important physiological function of APP in the organization of synapses and other cellular structures, although the molecular mechanisms of this process are not yet fully understood.

Aβ oligomers formed during APP cleavage, can form pore-like structures with channel activity that leads to damage to synapses [26,27]. In addition, Aβ can affect glutamate receptors [28], cause mitochondrial dysfunction [29,30], lysosomal insufficiency (reviewed in [31]) and changes in signaling pathways associated with synaptic plasticity, neuronal cells and neurogenesis [32,33].

Many studies in the field of understanding the functions of Aβ appear almost daily. Nevertheless, we still have little idea of how to look for approaches to the qualitative therapy of neurodegenerative diseases associated with amyloid accumulation. Perhaps it is after the discovery of native APP functions that an understanding of the occurrence mechanisms, timely diagnosis and prevention of these diseases will appear.

### 2.2. Tau-Protein

The cause of some dementias is considered to be tauopathy. Tau protein was first isolated from cells together with tubulin. It turned out that this protein can stimulate the assembly of microtubules [34]. Normally, tau protein is localized mainly in the axons of neurons, where it modulates the stability and assembly of microtubules necessary for axon growth and efficient axonal transport. Tau protein is also present in astrocytes and some glial cells [35]. In taupathies, neurofibrillary tangles (NFT) are formed from the insoluble hyperphosphorylated tau protein. This disorder is observed in patients with AD, PD, frontal temporal dementia, Pick’s disease, and others [3].

The tau protein, originally defined as MAP (microtubule-associated protein), has over time been discovered to possess many alternative functions. It has been shown to bind to the p150 subunit of the dynactin complex, which provides the interaction of dynein with cargo [36]. Some studies provide evidence that tau protein can interact with actin and affect its polymerization, as well as its interaction with microtubules [37,38,39]. Tau protein can also interact with the plasma membrane [40,41,42,43], with some proteins involved in signal transmission [44,45], and has a binding site to DNA and RNA [46,47].

The wide range of tau protein interactions with other proteins and cellular structures presents ample opportunities for research activities that may lead to the discovery of new treatments for neurodegenerative processes.

### 2.3. α-Synuclein

α-Synucleinopathies are the cause of dementia and, in particular, PD. In its native state, α-synuclein is a soluble protein consisting of 140 amino acid residues. Due to the central hydrophobic region, α-synuclein has a high tendency for aggregation. It forms an intermediate ring structure called a protofibrill, which eventually transforms into insoluble polymers or fibrils [48]. These insoluble fibrils are the main component of Lewy bodies, the accumulation of which is observed in the neurons’ cytoplasm in dementia, including PD. Lewy bodies are found in both sporadic and hereditary forms of this disease. It has been shown that the aggregation of α-synuclein can be influenced by many factors, such as mutations, post-translational modifications of the protein and even the dopamine effects [49,50].

Despite many studies, the exact function of α-synuclein remains unclear. Initially, this protein was detected in presynaptic endings [51], however, further studies have demonstrated its presence in other human tissues, including erythrocytes, in human and mouse cerebrospinal fluid, and even in cultured cells [52,53,54]. It also turned out that α-synuclein is contained in amyloid plaques in AD [55]. Data obtained on various models indicate the participation of α-synuclein in vesicular transport between the endoplasmic reticulum and the Golgi apparatus, and retrograde transport between endosomes and the Golgi apparatus [56,57]. A direct interaction between α-synuclein and the SNARES complex has been shown, as well as an effect on this complex assembly disruption in mice [58,59], which suggests the role of α-synuclein in the SNARES complex assembly. Thus, monomeric α-synuclein plays significant roles in synaptic signal transduction and synaptic vesicle recirculation [60], including influencing glutamate release [61]. In addition, this protein binds to F_0_-F_1_-ATPsynthase also affects ATP production [62].

From all of the above, it follows that α-synuclein performs a number of important functions in the cell and affects various cellular organelles. However, researchers have yet to determine its main role in normal cellular metabolism.

### 2.4. Prions

Prions (PrP) are unique pathogenic proteins that have the property of self-replication when acquiring an alternative conformation. Scrapie prion protein (PrP^Sc^) is the most studied mammalian prion. It is formed from the normal cellular prion protein (PrP^C^) by either spontaneous conversion or dominant mutations in the PRNP gene [63,64]. Prion diseases, or transmissible spongiform encephalopathies, includes Creutzfeld–Jacob disease, Gerstmann–Sträussler–Scheinker syndrome, kuru, fatal insomnia, and variable protease-sensitive prionopathy. All these diseases are associated with the conformational transformation of the normal cellular PrP into the abnormal PrP^Sc^ [65] through a post-translational process during which PrP acquires a high β-sheet content [66] with subsequent accumulation in the brain and nervous tissue [67]. Sometimes the term “prion diseases” includes other neurodegenerative diseases, consisting of AD and PD, although they should rather be called “prion-like”, since the mechanism of their development is similar, but associated with “prion-like” proteins, and not with PrP^Sc^.

PrP^C^ is a fairly conservative protein and is a sialoglycoprotein of 253 amino acids. It is encoded by a single gene and is found mainly on the cell surface [68]. Sometimes this protein is localized in the cytoplasm, exosomes and vesicles in a soluble form [69,70,71]. The participation of PrP^C^ in the development of prion diseases has been studied quite well, but its normal physiological role still remains the subject of scientific discussion. Conservatism and the expression of PrP^C^ in various animal and human tissues indicate the importance of this protein for normal cellular processes [72,73]. Apparently, PrP^C^ is involved during embryonic development, since its mRNA is detected both in germ cells and in various parts of the embryo’s nervous system [74]. Nevertheless, animals with PrP^C^ knockout develop and reproduce normally, which suggests the presence of some compensatory mechanisms [75]. According to accumulated data, it can influence some early processes of cell differentiation [76,77,78]. PrP^C^ plays a role in the development and maintenance of the nervous system as a whole [79,80,81]. The expression of PrP^C^ in various immune cells has been shown [82,83]. There is evidence of the importance of PrP^C^ in some types of cancer [84,85,86], as well as in the protection against oxidative stress during aging [87,88]. PrP^C^ plays a role in the growth kinetics of Aβ fibrils, reducing their aggregation [89,90].

Thus, PrP^C^ can play an active role in many different cellular processes and can influence a wide range of physiological phenomena in the body. However, to date, its exact function and relationship with many intracellular structures remains unexplored.

### 2.5. TAR DNA-Binding Protein 43

TAR DNA-binding protein 43 (TDP-43) is a highly conserved heterogeneous nuclear ribonucleoprotein that controls the transcription, splicing and RNA stability of certain genes (reviewed in [91]). This protein is involved in various aspects of cell proliferation and apoptosis [92]. Transcription of TDP-43 was found in various tissues. Normally, TDP-43 is localized mainly in the cell nucleus, but can circulate between the nucleus and the cytoplasm [93]. TDP-43 proteinopathy has been found in such forms of dementia as frontotemporal lobar degeneration and amyotrophic lateral sclerosis [93,94]. In these diseases, various forms of TDP-43 accumulate in the cytoplasm [94]. There is evidence of the presence of such clusters in AD [95], Lewy body disease [96] and Guam parkinsonism-dementia complex [97]. With the development of these diseases, the TDP-43 nuclear fraction is depleted, as well as a number of other RNA-binding proteins [98,99]. It remains unclear what causes neurotoxicity in this case, whether it is the aggregates themselves or the lack of regulatory factors for nucleic acids. It is apparent that both the loss of the TDP-43 native function and the toxicity of protein aggregates are important. This example shows that such combinations can be of great importance for the development of other neurodegenerative pathologies associated with the accumulation of Aβ, huntingtin, α-synuclein, etc.

### 2.6. Mutant Proteins in Polyglutamine Diseases

Currently, a number of neurodegenerative diseases have been described that have hereditary genetically determined causes, for example, polyglutamine diseases. This group of polyglutamine diseases, caused by the expansion of trinucleotide cytosine–adenine–guanine (CAG), repeats in the coding segment of certain genes. Such expansion leads to the appearance of mRNA with abnormally long repetitive CAG triplets and respective proteins with polyglutamine tracts in the cells. Today, a total of nine polyglutamine disorders (spinocerebellar ataxias (SCA) types 1, 2, 3, 6, 7, 17 [100,101,102,103,104,105,106]; dentatorubral–pallidoluysian atrophy; spinal and bulbar muscular atrophy [107]), have been described, and HD is one of them [5]. 

HD is the most studied among the polyglutamine neurodegenerative diseases. Its development is associated with a mutation in the *HTT* gene and the consequence of this mutation and defective huntingtin (HTT) expression, is the death of neurons, mostly striatal neurons. 

It has been shown that HTT is associated with numerous partner proteins, interacts with many intracellular structures and is involved in many cellular processes; therefore, HD is a very perspective model for the investigation.

Modern studies have been conducted, describing ALS patients with a pathological increase in CAG repeats in the *HTT* gene. At the same time, there is no striatum atrophy characteristic of HD, although such patients have a family history of HD cases with typical symptoms for this disease [108,109]. Meanwhile, there are reports of the TDP-43 and HTT aggregates in cells, probably arising from the direct interaction between the HTT polyglutamine fragment and the TDP-43 C-terminal domain, as well as the loss of motor neurons in patients with HD [110]. On the other hand, studies have shown that ALS manifestations are often found among patients with diagnosed HD [111]. These data allowed this study to hypothesize that various degenerative disorders, such as ALS and HD, may have a common genetic basis.

## 3. Huntingtin–Protein Interactions as the Basis of Intracellular Pathologies and Potential Target for Therapeutic Intervention in Huntington Disease

HTT is a major cellular protein. As one of the research teams aptly said, huntingtin is ‘here, there, everywhere’ [112]. The *HTT* gene expresses in all tissues and organs in humans and mice, but HTT expression level is the highest in neural tissue [112]. Autosomal dominant mutation in the HTT causes an increase in the polyglutamine fragment length (encoded by the CAG codon) at the protein N-terminus and leads to the development of HD, a severe and currently incurable neurodegenerative pathology [5,113,114].

**Figure 1:**
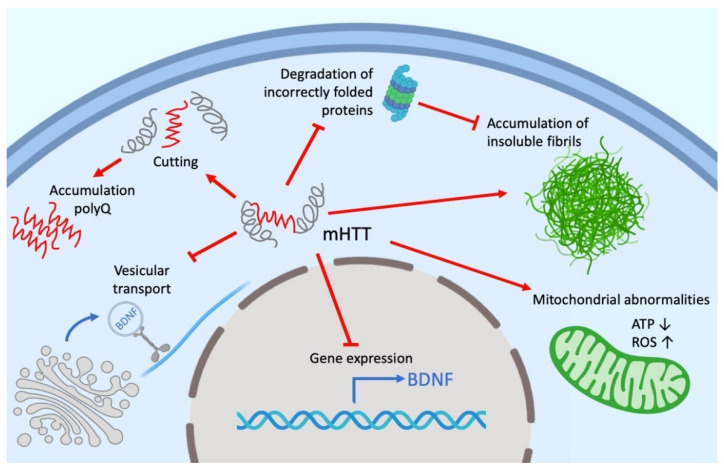
Possible mechanisms of mHTT cellular toxicity (enlarged polyglutamine fragment is shown in red). The toxic effect includes inhibition of proteasome and autophagic degradation, disruption of vesicular transport, effects on mitochondrial physiology, transcription of BDNF (brain-derived neurotrophic factor) and mitochondrial proteins. Polyglutamine fragments obtained during the degradation of mHTT itself also have a toxic effect. Red arrows indicate a stimulating effect, red T sticks indicate an inhibitory effect. Figure was created with BioRender.com (accessed on 25 October 2022).

Despite intensive investigations, the functions of both mutant (mHTT) and wild-type HTT remain not fully understood. HTT can play an active role in cell physiology, being involved in a wide range of intracellular processes, such as cell transport, endocytosis, protein degradation and other cellular and molecular processes. HTT, as well as mHTT, interacts with different cell organelles directly or through their associated proteins (Figure 1). It has been shown that HTT controls both anterograde and retrograde transport in organelles and neurotransmitters in axons, and dendrites of neurons [115,116,117,118]. It is assumed that the effect of HTT on transport is due to its interaction via HAP1 (huntingtin-associated protein 1) with the p150^Glued^ dinactin subunit and the kinesin family protein KIF5C [119,120]. HTT also interacts with both N- and C-terminal domains with dynamite 1 [112,121,122]. Our own experimental data give reason to believe that a significant part of the HTT-involved cellular processes is mediated by microtubules and other cell cytoskeleton structures [123,124]. HTT is involved in autophagy (reviewed in [125,126]). This protein may be a participant in the transcription regulation [127,128], the regulation of mitotic cell division [129] and the primary cilia formation [130,131,132].

According to many studies, the number of HTT partner proteins can range from 100 to 350 [121,133,134]. The large number of molecular interactions, the nature of partner proteins, the relatively large size and stability of HTT suggest its role as a scaffold for a variety of protein complexes. This is also evidenced by the presence of sites in HTT such as HEAT domains. Thus, HTT function may be to coordinate cellular processes as a central component of several protein complexes [135].

It was suggested that HTT interacting proteins are genetic modifiers in neurodegenerative disorders [121]. These authors have shown that HTT interacting proteins, confirmed as modifiers of the neurodegeneration phenotype, perform a wide range of biological functions, including synaptic transmission, cytoskeletal organization, signal transduction, and transcription. During the study, among the modifiers, 17 loss-of-function suppressors of neurodegeneration were found; these can be considered potential targets for therapeutic intervention.

## 4. The Spectrum of Possible Cellular Pathologies in Neurodegenerative Diseases

Experimental data indicate the involvement of many cellular processes in the neurodegenerative diseases development. These processes can be both unique to one disease and characteristic of many neurodegenerations. There are many discussions in the literature about phenomena such as the accumulation of protein aggregates or toxic protein fragments, violation of protein proteolysis, mRNA toxicity, mitochondrial disorders, nuclear violations, DNA damage, changes in protein expression and disruption of cellular transport. An attempt to compare and generalize these phenomena is given in Table 1.

The common manifestations of neurodegenerative diseases also include activation of microglia, cytokines, reactive astrogliosis and the launch of a broad inflammatory immune response [136,137]. These processes at the level of the organism lead to biochemical and structural changes in the surrounding neurons.

**Table 1:** Neurodegenerative diseases, proteins and cellular disorders, involved in neurodegeneration development.

Disease	Involved Proteins	Cellular and Intracellular Disorders
Alzheimer’s disease (AD)	APP and Aβ Tau	Impaired of protein folding and degradation [138]
		Lysosomal insufficiency (reviewed in [31])
		Mitochondrial dysfunction [29,30]
		Violation of signaling pathways
		Changes in protein expression [139,140]
		Synapses damage [26,27]
		Altered microtubule stabilization [141]
Parkinson’s disease (PD)	α-Synuclein Tau Parkin	Impaired of protein folding and degradation [142]
		Mitochondrial dysfunction (reviewed in [143])
		Endoplasmic reticulum stress [144]
		Cellular transport disorders (reviewed in [145])
Amyotrophic lateral sclerosis	TDP-43 SOD1 FUS	Mitochondrial dysfunction [146,147]
		Violation of axonal transport [148]
		Changes in RNA metabolism [149]
		Impaired protein degradation and autophagy [150]
Frontal temporal dementia	Tau TDP-43 FUS	Changes in gene expression [151]
		Endosomal trafficking dysregulation [152]
		Altered microtubule stabilization [153]
		Impaired protein degradation and autophagy (reviewed in [154])
Prion diseases	PrP^C^ and PrP^Sc^	Mitochondrial dysfunction [155,156]
		Endoplasmic reticulum stress [156]
		Impaired protein degradation [157]
		Synapses damage [158]
Huntington’s disease (HD)	HTT	Violation of protein folding and degradation [159,160]
		mRNA toxicity [161]
		Accumulation of toxic polyglutamine fragments [160,162,163]
		Cellular transport disorders (organelles, vesicles, neurotransmitters) [115,116,164]
		Violation of autophagy [165,166,167]
		Violation of signaling pathways [168,169]
		Mitochondrial dysfunction [170,171]
		Mitotic disorders [172]
		Transcription disorders [163]
		Violation of the primary cilia formation [130]

**Figure 2:**
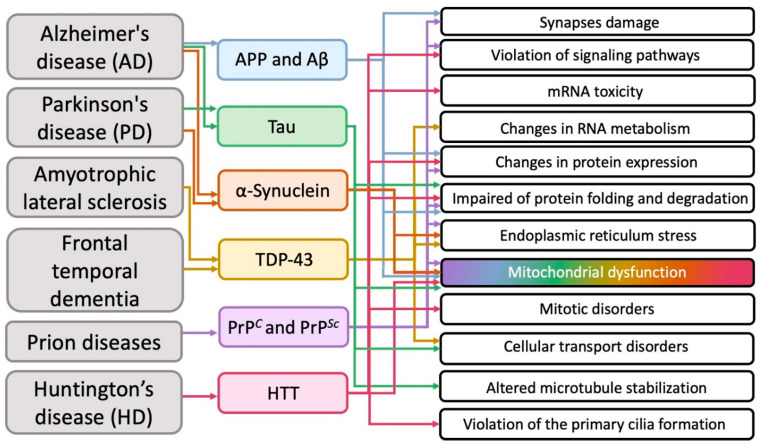
The main cellular pathologies developing during neurodegeneration.

Summarizing numerous experimental data (but, of course, without claiming to cover them completely), we tried to determine which intracellular structures and cytophysiological processes mainly undergo pathological changes during the development of the most common neurodegenerative diseases (Figure 2).

It transpired that in various neurodegenerative diseases on the subcellular structural level, in addition to the obvious general problems with the corresponding proteins (impaired of protein expression, folding and degradation), mitochondria most often suffer from (Figure 2).

Mitochondrial disorders are the most frequent in neurodegenerative diseases and are observed in both hereditary and sporadic forms. Excessive formation of free radicals, decreased ATP production, and damage to mitochondrial DNA are the most common mitochondrial disfunctions. It is these injuries that often become targets for the development of therapeutic approaches (for instance, the use of antioxidants), and such approaches are universal for neurodegenerative diseases. Thus, drugs aimed at maintaining mitochondrial function, for example, mitochondrial-targeted antioxidants, can be an essential part of the treatment of all neurodegenerative proteinopathy disorders.

The biological aspects of the influence of proteins involved in neurodegeneration remains a subject of debate. It is known that in AD Aβ inhibits some components of the respiratory chain, thus causing mitochondrial depolarization, and also causes an increase in calcium levels, which leads to oxidative stress [173,174,175]. Disruption of the Tau protein leads to an abnormal distribution of mitochondria [176], a violation of the balance of their fusion and division [177]. In addition, oxidative stress is also observed [178]. Binding of α-synuclein directly to the mitochondrial membrane has been demonstrated [179]. This causes inhibition of the respiratory chain complex I [180] and stimulation of the calcium signal [181]. mHTT leads to a decrease in the activity of some components of the respiratory chain and a decrease in the membrane potential of mitochondria, as well as the buffer capacity of calcium [182,183].

The transcription of some neurotransmitters genes is disrupted in polyglutamine diseases, and for the HTT protein, the direct effect upon gene transcription is evident. Additionally, in neurodegenerative diseases there is a violation of the transcription factors’ transport from the cytoplasm to the nucleus (reviewed in [184]). Most neurodegenerative diseases are characterized by post-transcriptional modification changes [185].

Recent experimental data have confirmed the effectiveness of alternative splicing modulators in eliminating aberrant mHtt-induced splicing. The potential to use these modulators as a method of successful HD therapy looks very promising [186].

Proteomic analysis in AD, PD and polyglutamine diseases demonstrates changes in the expression of proteins involved in energy metabolism, synaptic function, axon growth, chaperone activity and protein degradation (well described in review [187]). Such studies confirm the idea that neurodegenerative processes are based on common cellular disorders: accumulation of incorrectly folded proteins, dysfunction of the protein degradation system, damage to mitochondria, and transport disorders. There are studies devoted to the translation disturbances in neurodegeneration, with a decrease in the level of various translation factors, and the presence of mutations in some ribosomal proteins (reviewed in [188]). Thus, it seems that neurodegeneration causes disorders both at the level of transcription and translation, although further studies are required for detailed research of these aspects.

## 5. Modern Approaches to the Development of Methods for Neurodegenerative Diseases Treatment

Modern medicine commonly uses exclusively symptomatic therapy, which is not able to affect the dynamics of the pathological process. Therapeutic approaches are designed to eliminate the manifestations of neurological disorders and somatic disorders that have arisen as a result of the disease development. Treatment methods include drugs that affect the neurotransmitter component (cholinesterase inhibitors and glutamate regulators in AD; catechol O-methyltransferase (COMT) inhibitors, MAO-B inhibitors, anticholinergics, L-Dopa, dopamine agonists and MAO-B inhibitors in PD; dopamine antagonists in HD); various channel blockers in ALS and ataxia (neuroprotectors); antipsychotics; antioxidants; and sedatives and muscle relaxants for the relief of choreic symptoms in HD. The therapy used is not specific to certain forms of neurodegenerative diseases and nor have a specific point of application to the regulation mechanisms of a certain protein, as such a violation of the conformation or structure causes the disease development.

As it follows from the analysis of modern research data (Figure 2), mitochondria and protein degradation systems in cells are most often affected in neurodegenerative diseases. To date, it is obvious that such disorders are most likely not the cause, but the earliest consequence of the development of the neurodegenerative process. Since mitochondrial disorders and protein degradation anomalies that occur in this case are characteristic of all neurodegenerations, mitochondria and cellular systems involved in the degradation process can serve as a target for the development of new therapies, specifically aimed at restoring their normal functioning and protecting certain cellular organelles.

Examples of such approaches include trials of mitochondrial-targeted antioxidants, such as SkQ1 and MitoQ, against neurodegenerative diseases (well described in review [189]). Theoretically, this approach seems promising, but in laboratory studies, mitochondrial-targeted antioxidants have not yet been shown to significantly improve patients in clinical trials [190]. It is possible that the effectiveness of the use of mitochondrial antioxidants will be much higher if they are used in the early stages of the disease, before the development of pronounced damage to neurons. Therefore, the development of methods for the early diagnosis of neurodegenerative diseases is indeed an extremely urgent task.

In recent years, innovative immunological, genetic engineering and molecular biological approaches to the treatment of neurodegenerative diseases have been developed in the laboratory. One of the promising ways to combat the progressive development of neuron death is the vaccination of patients against neurodegeneration proteins (Aβ, α-synuclein etc.) [191], as well as the use of antibodies specific to them [192]. Theoretically, such an approach should be successful, but clinical trials conducted at the moment demonstrate its weak effectiveness for treatment; thus, its improvement and modification are required, apparently at the stage of preclinical trials. Methods aimed at editing mutant genes are being developed for the treatment of hereditary forms of neurodegenerative diseases. Researchers are looking for approaches to correcting the mHTT gene in HD using the CRISPR/Cas9 system [193]. At the same time, methods aimed at reducing the level of mutant proteins in the cell by affecting expression are being actively developed. These strategies have huge potential for treatment. RNA-targeted methods include the use of synthetic antisense oligonucleotides that bind to a specific sequence of ribonucleic acid, which can reduce the translation of mRNA into a disease-causing protein [194]. The use of the RNA interference mechanism shows satisfactory results in HD [195,196,197]. Thus, the specific effect on neurodegeneration-associated proteins in this case is the key to the development of effective therapies. Finally, an approach based on the use of neurodegeneration suppressor proteins, which can be considered as potential targets for therapeutic intervention, has already been mentioned in this review [114] and seems very promising.

Despite the theoretical validity of the innovative approaches described above, the numerous methods of treatment of neurodegenerative diseases being developed have a very significant drawback. The basis of their therapeutic concept is a more or less unique approach to the elimination of one specific molecular pathology. At the same time, the accumulated experimental data already indicate that the occurrence and progression of neurodegeneration is always associated with many different internal and external factors. Many neurodegenerative diseases may occur as a result of damage to not one, but several genes, as we see in the example of HD and ALS. In such cases, studies aimed at identifying the specific functions and unique properties of all involved neurodegeneration-associated proteins can help in the development of innovative therapies. Thus, it is undesirable to exclude an individual approach in the treatment of each disease and its subtype, but it must be combined with the general principles of therapy (for example, the use of antioxidants). Only in this case can targeted therapy give significant results in improving the condition of patients.

## 6. Biomarkers for Early Detection of Neurodegenerative Diseases

As follows from the attempts described above to create effective strategies for the treatment of neurodegenerative diseases, their inefficiency is often associated with the problem of late detection due to the lack of early diagnostic methods. When the process of neuronal death has already started and has led to the development of atrophic disorders in the nervous system, the most modern molecular approaches come to a dead end and do not bring tangible relief to patients. Thus, the efforts of scientists should be directed to the search for biomarkers that would allow the determination of the risks of neurodegeneration in patients before the symptoms of the disease. This task is not solved at the moment, but work in this direction is underway and researchers offer a wide variety of approaches. For example, to detect AD in the early stages, the use positron emission tomography, which detects even a small accumulation of Aβ [198], has been proposed. It has been shown that in some neurodegenerative diseases, there is a change in the level of various forms of neurodegeneration-associated proteins in the CSF, and changes can be recorded long before the appearance of protein aggregates [199,200,201]. CSF sampling is a serious and invasive procedure, so detecting the first signs of protein disorders in more easily accessible fluids and tissues would be a big step for-ward. The search for such lightweight approaches is focused on application in the diag-nosis of peripheral tissues [202]. Biomarkers of neurodegenerative diseases should be available for detection in those biological samples, the sampling of which is easy to carry out during routine medical examination procedures.

Alternative approaches to early diagnosis are also described in the literature, for example, muscle action potential measurement methods. A simple physical method of muscle action potential measurement in ALS was proposed for practical application as a biomarker for longitudinal follow-up and clinical trials in ALS [203].

## 7. Conclusions

A very urgent problem of modern medicine is the study of mechanisms, and the search for new ways to compensate for damage and protect neurons in chronic neurodegenerative diseases of the central nervous system, since they are currently incurable. The methods of symptomatic treatment used in clinics make it possible to alleviate the condition of patients to some extent and slow down the development of neurodegenerative processes, but they cannot bring the disease under control or noticeably prolong the patient’s life.

Despite the huge amount of research being conducted, many fundamental questions have remained unanswered until now. In particular, the native functions of many proteins participating in the neurodegenerative process and the trigger mechanisms provoking the onset of the disease are not characterized at the molecular level. That is why modern researchers’ interest in the mechanisms of the development of neurodegenerative processes is often aimed at studying the native functions of proteins, whose mutant forms are involved in the pathogenesis of neurodegenerative diseases. It is possible that here lies the key to the diagnosis and prevention of neurodegeneration in the early stages.

The compilation of the available experimental data on morphological and functional pathologies of intracellular structures characteristic of the most common neurodegenerative diseases allows us to conclude that the most vulnerable cellular organelles are mitochondria (Figure 2). Thus, therapy aimed at restoring and maintaining mitochondrial function can already be offered as a general approach to the treatment of a certain group of neurodegenerative diseases. In this case, mitochondrial-targeted antioxidants can be used as a therapeutic agent.

Obviously, it is impossible to offer a universal approach to the treatment of all neurodegenerative diseases. It is necessary to search for individual approaches to protect the structure and functions of specific proteins involved in the development of neurodegeneration at the cellular level. In this regard, promising are the studies conducted on the HD model, allowed to detect HTT-interacting proteins that are genetic modifiers in neurodegenerative disorders [121]. Non-functioning neurodegeneration suppressors found among modifiers can be considered potential targets for therapeutic intervention. The search for similar neurodegeneration suppressor proteins, among partner proteins interacting with mutant proteins involved in the development of other neurodegenerative processes, seems to be a promising direction for further research aimed at restoring the normal function of damaged proteins.

Genetic engineering approaches aimed at editing mutant genes [193], strategies aimed at reducing the level of mutant proteins in the cell [194], vaccination of patients against neurodegeneration proteins (Aβ, α-synuclein etc.) [191], and the use of specific antibodies to them [192], seem very promising. Having appeared not so long ago, these approaches are actively developing and in the future may ensure the emergence and introduction of fundamentally new methods of treatment of neurodegenerative diseases into therapeutic practice.

**Figure 3:**
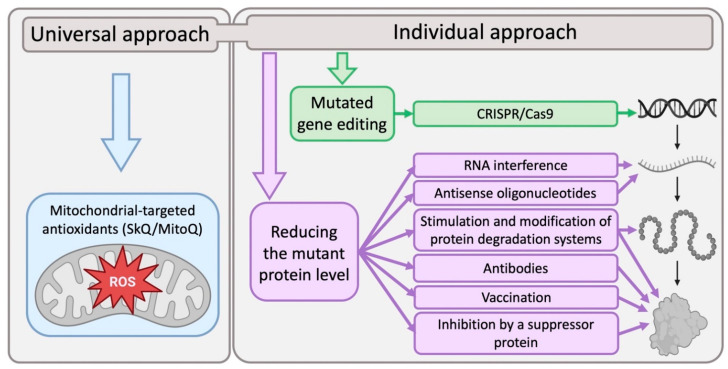
The proposed strategy to the neurodegenerative diseases treatment. The most promising strategy looks at a combination of universal mitochondrial therapy and one of the innovative individual approaches for each disease. Figure was created with BioRender.com accessed on (accessed on 25 October 2022).

Thus, a combination of therapy aimed at maintaining mitochondrial function and any of the above innovative approaches aimed at editing mutant genes, at suppressing (by means of neurodegeneration suppressor proteins), or reducing the level of mutant proteins in the cell (for diseases that are not the result of a genetic mutation) (Figure 3), currently seems to be the most promising approach for the treatment of these disorders. In our opinion, taking into account the current state of research, the greatest progress regarding these approaches should be expected in areas related to the development of methods aimed at reducing/suppressing the level of mutant/defective proteins in the cell. They can provide a qualitative breakthrough in the treatment of neurodegenerative diseases.

## Data Availability

Not applicable.
